# The Cost and Cost-Effectiveness of Scaling up Screening and Treatment of Syphilis in Pregnancy: A Model

**DOI:** 10.1371/journal.pone.0087510

**Published:** 2014-01-29

**Authors:** James G. Kahn, Aliya Jiwani, Gabriela B. Gomez, Sarah J. Hawkes, Harrell W. Chesson, Nathalie Broutet, Mary L. Kamb, Lori M. Newman

**Affiliations:** 1 Philip R. Lee Institute for Health Policy Studies, University of California San Francisco; Super Models for Global Health, Berkeley, California, United States of America; 2 Super Models for Global Health, Arlington, Virginia, United States of America; 3 Department of Global Health, Academic Medical Centre, University of Amsterdam and Amsterdam Institute for Global Health and Development, Amsterdam, The Netherlands; 4 UCL Institute for Global Health, University College London, London, United Kingdom; 5 Division of Sexually Transmitted Disease Prevention, Centers for Disease Control and Prevention, Atlanta, Georgia, United States of America; 6 Department of Reproductive Health and Research, World Health Organization, Geneva, Switzerland; Hopital Bichat Claude Bernard, France

## Abstract

**Background:**

Syphilis in pregnancy imposes a significant global health and economic burden. More than half of cases result in serious adverse events, including infant mortality and infection. The annual global burden from mother-to-child transmission (MTCT) of syphilis is estimated at 3.6 million disability-adjusted life years (DALYs) and $309 million in medical costs. Syphilis screening and treatment is simple, effective, and affordable, yet, worldwide, most pregnant women do not receive these services. We assessed cost-effectiveness of scaling-up syphilis screening and treatment in existing antenatal care (ANC) programs in various programmatic, epidemiologic, and economic contexts.

**Methods and Findings:**

We modeled the cost, health impact, and cost-effectiveness of expanded syphilis screening and treatment in ANC, compared to current services, for 1,000,000 pregnancies per year over four years. We defined eight generic country scenarios by systematically varying three factors: current maternal syphilis testing and treatment coverage, syphilis prevalence in pregnant women, and the cost of healthcare. We calculated program and net costs, DALYs averted, and net costs per DALY averted over four years in each scenario. Program costs are estimated at $4,142,287 – $8,235,796 per million pregnant women (2010 USD). Net costs, adjusted for averted medical care and current services, range from net savings of $12,261,250 to net costs of $1,736,807. The program averts an estimated 5,754 – 93,484 DALYs, yielding net savings in four scenarios, and a cost per DALY averted of $24 – $111 in the four scenarios with net costs. Results were robust in sensitivity analyses.

**Conclusions:**

Eliminating MTCT of syphilis through expanded screening and treatment in ANC is likely to be highly cost-effective by WHO-defined thresholds in a wide range of settings. Countries with high prevalence, low current service coverage, and high healthcare cost would benefit most. Future analyses can be tailored to countries using local epidemiologic and programmatic data.

## Introduction

Syphilis in pregnancy is an under-recognized problem that carries a significant public health and economic burden. Each year, nearly 1.5 million pregnant women around the world are infected with probable active syphilis [1]. According to a recent meta-analysis [2], over half of untreated pregnancies affected by syphilis result in adverse outcomes known collectively as mother-to-child transmission (MTCT) of syphilis. These include stillbirth and second or third trimester fetal loss (21%), neonatal death (9%), premature and low birth-weight infants (6%), and infants with clinical syphilis infection (16%) [2]. The current global burden of disease attributable to MTCT of syphilis is estimated at 3.6 million disability-adjusted life years (DALYs), comparable to MTCT of HIV [1]. Annual global direct medical costs for MTCT of syphilis total an estimated $309 million [1].

Screening and treatment for syphilis in pregnancy is relatively simple and inexpensive, and has been shown to be cost-effective even in low-resource settings [3]–[7]. New technologies, such as point-of-care tests, can be used regardless of limited infrastructure and allow for diagnosis and treatment at a single visit [8]. Treatment with penicillin is inexpensive, and if diagnosed early, is highly effective in preventing adverse pregnancy outcomes associated with syphilis in pregnancy [9].

Despite this promising potential for intervention, screening and treatment of syphilis in pregnancy is not yet universal. Policies on screening exist in many countries, but partial or poor programmatic implementation means that more than 60% of pregnant women do not receive screening, or receive it too late for treatment to be effective [10], [11]. To address this problem, in 2007, World Health Organization (WHO) began an initiative to eliminate global MTCT of syphilis, promoting the fundamental elements of advocacy, political commitment, as well as monitoring, scale-up and integration of syphilis interventions into existing antenatal care (ANC) and prevention of MTCT of HIV programs [12]. The initiative aims to ensure that at least 90% of pregnant women are screened for syphilis, and 90% of those identified with syphilis are treated appropriately.

The objective of this study was to model the incremental cost-effectiveness of scaling-up syphilis screening and treatment in existing ANC programs. We examined systematically varied country case scenarios in order to portray results for diverse programmatic, epidemiologic, and economic contexts.

## Methods

### Ethics statement

An ethics statement was not required for this work.

### Overview

Using a Microsoft Excel model, we analyzed the cost, health impact, and cost-effectiveness of expanded syphilis screening and treatment in antenatal care, compared to the current level of services, for eight generic country case scenarios. We examined cohorts of 1,000,000 pregnancies per year, for four years. The four-year time frame was chosen as it represents WHO's proposed initial duration of intensified support for MTCT of syphilis elimination of countries, though we would hope that these efforts would continue past these four years. For the cost analysis, we assessed the direct medical costs of implementing expanded testing and treatment of syphilis in pregnancy. For the health impact analysis, we estimated the health benefits of an expanded program in terms of averted clinical adverse outcomes and DALYs. The cost-effectiveness analysis was adjusted for offsetting savings due to replaced programming and averted adverse outcomes, and calculated incremental cost-effectiveness ratios as the cost per DALY averted. We used a societal perspective; discounted long term costs and DALYs at 3% per year; and expressed results in 2010 US dollars. Table 1 summarizes input assumptions.

**Table 1:** Base Case Inputs and Assumptions.

	BC value*			
Input	LO	HI	One-way SA range**	Source/Notes
Cohort size	1,000,000		n/a	Assumption
Discount rate	3%		n/a	
Pr. active syphilis in women with a reactive syphilis serological test	65%		50% – 80%	[17]–[19]
Pr. reactive syphilis serological test in pregnant women	0.5%	3.0%	HI: 3.0% – 6.0%	Assumption
Test sensitivity of RPR	100%		70.7 – 100%	[34]
Current % tested and treated	20%	70%	LO: 10% – 40% HI: 50% – 72%	Assumption
Health service cost level (inpatient)	0.25	1	LO: 0.10 – 0.33	WHO CHOICE [16]
Health service cost level (outpatient)	0.75	1	LO: 0.20 – 0.75	WHO CHOICE [16]
**Intervention characteristics**				
% Attending ANC	70%	95%	LO: 60% – 80% HI: 90% – 99%	Assumption
% Screened	80%	90%	LO: 70% – 90% HI: 85% – 99%	Assumption
% Treated	90%	95%	LO: 80% – 95% HI: up to 99%	Assumption
Treatment performance	90%		70% – 99%	[20]; decreasing efficacy with later treatment (Hawkes, unpublished)
**AO incidence (no intervention)**				
All AO	52%		40% – 70%	[2]
Stillbirth/2^nd^/3^rd^ trimester fetal loss	20.9%		16.2% – 28.3%	Proportional incidence
Neonatal death	9.3%		7.3% – 12.7%	Proportional incidence
Infected infant	15.5%		11.9% – 21%	Proportional incidence
Prematurity or low birth weight	5.8%		4.6% – 8%	Proportional incidence
Adult syphilis averted per syphilis positive pregnancy treated	1		0 – 1	Assumption
HIV cases averted per syphilis positive pregnancy treated	0.001		0 – 0.001	[24]
**DALYs**				
Stillbirth/2^nd^/3^rd^ trimester fetal loss	4.95		0 – 30	[22]; Assumption
Neonatal death	9.4		0 – 30	[22]; Assumption
Infected infant	9.48		6 – 15	[22]; Assumption
Prematurity or low birth weight	3.18		1.59 – 4.77	[22]; Assumption
Adult STI (HIV and syphilis)	1.34		0.67 – 2.01	[22]; Assumption
HIV	7.2		4.75 – 9.5	[35]
**Costs** †				
Stillbirth/2^nd^/3^rd^ trimester fetal loss	$0	- $1?	n/a	[6]
Neonatal death	$893	$3,571	n/a	[6]
Infected infant	$182	$243	n/a	[6]
Prematurity or low birth weight	$366	$1,464	n/a	[6]
Primary syphilis	$15	$20	n/a	Based on single visit, test, PCN
Secondary & early latent syphilis	$15	$20	n/a	Based on single visit, test, PCN
Late latent & tertiary syphilis	$500	$2,000	n/a	U.S. est. [36] adjusted for lower price and incidental treatment in developing countries and inpatient unit costs [16]
HIV infection	$6,500		n/a	[35]
Syphilis test with labor & supplies	$1.83	$2.30	LO: $1.48–$2.22 HI: $1.82–$2.56	WHO Bulk Procurement and IDA Foundation estimates (unpublished data, 2012) [6]
Course of benzathine penicillin (3 doses) including counseling	$3.72	$3.79	LO: $1.39–$3.72 HI: $1.46–$3.79	WHO Bulk Procurement estimates (unpublished data, 2012); [6]

* Low and high values of the base case are provided for inputs that vary based on the country case scenario. Each case scenario is characterized by low or high values on three factors: the prevalence of a reactive syphilis serological test in pregnant women, the percentage of women tested and treated for syphilis in ANC at the current level of services, and the relative cost of health services (including the cost of PMTCT of syphilis AOs). Accordingly, the percentages of women attending ANC and tested and treated for syphilis under the expanded program vary by case scenario, as do the costs of syphilis AOs and of screening and treating syphilis in the mother. All other base case inputs are constant across the eight case scenarios.

** Two sensitivity analysis ranges (high and low) are provided for inputs that vary based on the country case scenario. For all other inputs explored in SA, a single range is given

† For each MTCT of syphilis AO we estimate the cost by subtracting the cost of the healthy childbirth from the cost of the AO; this might be overestimate the benefits of preventing MTCT of syphilis as it assumes that all infants would otherwise be born healthy.

? Negative costs imply savings. Based on published data from South Africa, the cost of delivery of a stillborn infant was assumed to approximate the cost of delivery of a healthy infant (i.e., $58), and the cost of a spontaneously aborted pregnancy was ∼$57). We estimated the cost of a stillbirth/2^nd^/3^rd^ trimester fetal loss as the cost of a spontaneously aborted pregnancy minus the cost of a normal delivery (what the cost would have been in the absence of the AO), i.e., −$1. In settings where the cost of health care services is high, the estimate is −$1, and in those where the cost of services is low, the estimate is $0 (because of the adjustment for the cost per hospital day).

### Country case scenarios

Based on published and empirical evidence, we generated plausible generic country scenarios in order to describe results for a wide range of potential intervention settings. This approach avoids the complexities and nuances of local data, which we believe are best addressed as part of individual country analyses rather than a global view.

We selected three contextual factors predictive of the incremental cost and impact of scaled-up syphilis screening and treatment: current syphilis testing and treatment coverage in ANC, prevalence of syphilis among pregnant women attending ANC, and the relative cost of health services. We assigned plausible high and low values to each factor. Varying these three dichotomous factors together produced eight combinations and thus eight case scenarios, e.g., Country A: high current service coverage, high prevalence, and high cost; Country B: low current service coverage, high prevalence, and high cost; etc. The scenarios are summarized with the results in Table 2. Using publicly available data on ANC syphilis prevalence [13], ANC syphilis testing [14], and health care service costs [15], we created a table matching countries to each of the eight scenarios in order to help contextualize the generic scenarios (Table S1).

**Table 2:** 4-year cost-effectiveness of the MTCT of syphilis elimination program compared to current screening and treatment in 8 country scenarios.

Country scenario*					Cost-effectiveness findings **			
Scenario	Syphilis prevalence in ANC	Current ANC screening & treatment coverage	Cost of health services	DALYs averted (4 years)	Cost of intervention (4 years)	Offsetting savings***(4 years)	Net cost/savingŝ	Cost per DALY averted
**A**	High	Low	Low	93,484	$4,329,722	$6,272,739	−$1,943,017	<$0
**B**	High	Low	High	93,484	$5,381,458	$17,642,708	−$12,261,250	<$0
**C**	High	High	Low	34,518	$6,629,636	$7,395,199	−$765,563	<$0
**D**	High	High	High	34,518	$8,235,796	$12,823,574	−$4,587,778	<$0
**E**	Low	Low	Low	15,584	$4,142,287	$2,405,480	$1,736,807	$111
**F**	Low	Low	High	15,584	$5,190,243	$4,646,771	$543,472	$35
**G**	Low	High	Low	5,754	$6,327,564	$5,734,376	$593,188	$103
**H**	Low	High	High	5,754	$7,927,633	$7,787,351	$140,282	$24

Costs are in 2010 USD

* Scenario classifications are: prevalence of syphilis  =  high (3%) or low (0.5%), current ANC screening and treatment coverage  =  high (70%) or low (20%), cost of health services reflects assumptions about the overall relative health care cost structure, including the cost of MTCT of syphilis AOs, i.e. high (1) or low (0.25) based on WHO CHOICE data (http://www.who.int/choice/en/).

** Cost and effectiveness compared to current screening and treatment services

*** Offsetting savings  =  costs of net averted MTCT of syphilis and net averted adult STI plus costs of current services replaced by expanded program

? Savings denoted by negative costs

The high and low values for each factor were defined as follows; further variation around the scenario-defining values is examined in sensitivity analyses:

The current coverage of syphilis testing and treatment in ANC reflects the percentage of women in ANC who receive both testing and treatment to prevent MTCT of syphilis. We varied this factor from a low of 20% to a high of 70% [1] to reflect regional variations in current ANC capacity, syphilis screening policies, and implementation of those policies [10].

The prevalence of syphilis among pregnant women attending ANC was defined as the percentage of women attending ANC with a reactive test result based on a syphilis serological test. We varied this factor from a low of 0.5% to a high of 3% [1].

We defined the cost of health services as the relative cost of comparable health services based on WHO CHOICE unit cost data [16]. For inpatient care, we specified the relative unit cost differential between the country for which we obtained cost data (South Africa)[6] and lower cost countries as 1 to 0.25. For outpatient care, the analogous ratio in WHO CHOICE is 1 to 0.75.

### Inputs and assumptions

#### Burden of disease: MTCT of syphilis adverse outcomes in the absence of syphilis screening or treatment

From the literature, approximately 65% of pregnant women with reactive syphilis serologic tests have probable active syphilis, i.e., infections with reactive treponemal as well as non-treponemal (e.g., RPR) serologic tests, suggesting potential for MTCT of syphilis to occur [17]–[19]. Thus, we calculated a prevalence of active syphilis of 0.33%–1.95% across the scenarios. A recent meta-analysis estimated that 52% of pregnancies among women with untreated active syphilis infection result in adverse outcomes for the infant caused by syphilis [2]. Therefore, for 1,000,000 pregnancies per year in our analysis, in the absence of syphilis screening or treatment an estimated 1,697 MTCT of syphilis adverse outcomes (AOs) would be expected in low syphilis prevalence settings, and 10,179 AOs in high syphilis prevalence settings (6,786 – 40,716 AOs over four years).

#### Burden of disease: MTCT of syphilis adverse outcomes assuming current levels of screening and treatment

With partial implementation of syphilis screening and treatment in ANC (20% in low and 70% in high coverage settings), and a treatment efficacy of 90% (based on published evidence,[9], [20] and assuming a certain percentage of women receive treatment after the period of maximum efficacy), we estimated between 305 – 6,413 AOs would be averted per country per year with the current level of services.

#### Burden of disease: MTCT of syphilis adverse outcomes with expanded screening and treatment

Based on the proportion of pregnant women screened and treated at the current level of services, we made the following assumptions about an expanded screening and treatment program. In low coverage settings (currently 20% screened and treated), we assumed that 70% of pregnant women would attend ANC services, of whom 80% would receive syphilis testing, and 90% of those with a reactive test would receive treatment. Though the goals for the MTCT of syphilis elimination initiative are for 90% of pregnant women to be screened and 90% of syphilis reactive cases to be treated by 2015, we conservatively assumed a lower percentage would be screened in current low coverage settings, due to lower baseline levels of testing and treatment. In high coverage settings (currently 70% screened and treated), we assumed 95% of pregnant women attend ANC services, of whom 90% would receive testing, and 95% of those with a reactive test would receive treatment. Therefore, under the expanded program, these assumptions would translate to an increase from current coverage levels of 20% to 50.4% of women tested and treated in low coverage settings, and an increase from 70% to 81.2% in high coverage settings. In the base case, we assume an RPR sensitivity of 100%; however RPR sensitivity may vary based on the stage of syphilis at the time of diagnosis. We test the robustness of this assumption in sensitivity analysis.

We calculated the expected net AOs averted over four years as the product of the number of AOs in the absence of treatment and the increase in syphilis screening and treatment coverage from current to expanded services, assuming 90% treatment efficacy [9], [20]. Using published data on the rates of each AO in untreated pregnancies, we then calculated the numbers of each specific AO averted under the expanded program.

#### Burden of disease: DALYs

A DALY is a summary measure of disease burden that combines, for a specific disease or condition, the number of years of life lost due to premature mortality (YLLs) with that of years of life lost due to disability (YLDs) [21]. The numbers of DALYs associated with each AO were derived from estimates in the 2006 Global Burden of Disease [22], as described below.

We assumed a stillbirth causes 4.95 DALYs and a neonatal death causes 9.4 DALYs – conservative assumptions based on a 3% discount rate, uniform age weights, and a gradual acquisition of life potential (ALP; i.e., that deaths that occur at very young ages (<5 years) carry less weight than those that occur later in childhood, with the number of years of life lost gradually increasing from deaths occurring near the time of birth to deaths occurring at the age of 5) [23]. Though previous assessments of the global burden of disease have not included stillbirths, Jamison and colleagues have suggested the incorporation of ALP as a way to include and flexibly weight stillbirths and other early deaths when assessing disease burden [23].

We assumed an infant born with low birth weight and an infant infected with syphilis carry DALY burdens of 3.18 and 9.48, respectively, based on disability weights of 0.106 and 0.316, respectively [22], applied to the 30 discounted life year potential of a normal lifespan. We calculated the expected DALYs averted in fetuses and infants over the four years of the expanded program as the product of the number of each AO and the DALYs associated with that AO.

Given the dynamics of syphilis transmission, it is plausible that syphilis treatment may avert additional cases, though data are lacking on the number of cases averted per case of syphilis treated. In the base case, we assumed that each case of treated syphilis in pregnancy would avert one case of adult syphilis. In sensitivity analysis, we explored the effect on cost-effectiveness of a more conservative assumption, with modified estimates of 0.5 cases and 0 cases averted per case of syphilis treated (see Text S1 for details). Syphilis is a sexually transmitted infection, and infection with syphilis increases the risk of HIV transmission and acquisition [24], [25]. We assumed that treating syphilis in pregnancy has a modest effect in reducing syphilis-attributable HIV cases, and that treatment would avert 0.759 DALYs on average (the weighted mean DALYs from adult syphilis and HIV; see Text S1 for details).

The expected net DALYs averted over the four years of the MTCT of syphilis elimination program was calculated as the sum of the DALYs averted in fetuses and infants and those due to reduced STI in adults.

#### MTCT of syphilis elimination program cost

Under the new initiative, we assumed universal use of a syphilis serological test for screening and diagnosis of syphilis in ANC. We calculated the cost of the program for four years as the costs of testing (i.e., the syphilis test kit and the cost of labor and supplies), plus the costs of treatment for seropositive women (i.e., counseling and a three-dose course of penicillin). While a single dose of penicillin is sufficient to treat pregnant women with primary, secondary and early latent syphilis, three doses are recommended for the treatment of late latent syphilis [26], and we conservatively assumed universal use of a three-dose course. We explored the effect on costs of using a single dose of penicillin in a sensitivity analysis. The prices of a syphilis serological test (specifically, an RPR test) and a three-dose course of long-acting, intramuscular penicillin were determined from the WHO bulk procurement system, with allowances for delivery costs (WHO, unpublished data, 2012). The RPR test, with transport, was calculated as $0.21 (the cost of one test from a kit of 100) plus $0.21 (the cost of airfreight, packaging and insurance per test, based on an order of 2100 tests at $450 total transport costs). The three-dose course of penicillin was calculated based on a price of €34.82 per 50-vial box (converted to USD at 1.25 conversion), with 30% added cost for shipping, and $3.30 per box of 100 sterile water vials for reconstitution.

Though we used the cost of an RPR test in the base case analysis, use of a rapid diagnostic test (RDT) –another type of syphilis serological test– would have resulted in a very similar cost estimate when factoring in additional components such as transport, labor and supplies. The WHO bulk procurement system cost of the RDT including transport was calculated as $0.40 (the cost of one test from a kit of 25) plus $0.07 (the cost of airfreight, packaging and insurance per test based on an order of 10,000 tests at $740 total transport cost). Including labor and supplies[6] the total estimated cost of an RDT is $1.82 versus $2.30 for an RPR (based on WHO bulk procurement system quotes); using price quotes from the IDA Foundation, the total estimated cost of an RDT is $2.56 versus $2.02 for the RPR, yielding overlapping ranges for these two types of syphilis serological tests (RPR: $2.02 – $2.30; RDT: $1.82 – $2.56). We explored a plausible range of syphilis serological test costs in sensitivity analysis.

The costs of labor and supplies, and the cost of syphilis counseling were derived from an analysis using costs from South Africa[6]. Using WHO CHOICE unit cost data[16], we adjusted the costs of testing and treatment across country scenarios based on constant commodity costs (test kits and penicillin) and the relative cost of outpatient care (described above). The overall cost of testing across the scenarios was $1.83 – $2.30 per woman, and of treatment with a three-dose course of penicillin was $3.72 – $3.79.

#### Cost of AOs

The cost of each MTCT of syphilis AO was derived from two studies using costs from South Africa[5], [6], which estimated the cost of hospitalization for treatment. All costs were adjusted to 2010 US dollars using an inflation rate of 4% per year, and then further adjusted based on the cost level (cost per hospital day) in that country scenario (i.e. by 0.25 in low cost countries, and by 1.00 in high cost countries). The cost of delivery of a stillborn infant was assumed to approximate the cost of delivery of a healthy infant (i.e., $58), and the cost of a spontaneously aborted pregnancy was ∼$57). We estimated the cost of a stillbirth/fetal loss as the cost of a spontaneously aborted pregnancy minus the cost of a normal delivery (what the cost would have been in the absence of the AO), i.e., −$1. In settings where the cost of health care services is high, the estimate is −$1, and in those where the cost of services is low, the estimate is $0 (because of the adjustment for the cost per hospital day). For the estimate of the cost of treatment for an infant infected with clinical syphilis, we assumed that 30% of cases would be discovered and treated based on clinical findings.

#### Offsetting savings from MTCT of syphilis elimination program

While the program carries a cost for expanded screening and treatment, savings are accrued due to averted MTCT of syphilis AOs and averted adult sexually transmitted infections (STIs) (syphilis and HIV). Moreover, since at least a low level of maternal syphilis services are available in each of the country scenarios under consideration, we assumed that the prior services would be replaced by the new program and we adjusted the cost of the expanded program accordingly.

We calculated the savings due to net averted MTCT of syphilis AOs as the combined cost of each AO times the number of each AO prevented under the program.

We calculated the savings due to net averted adult syphilis and HIV cases as the number of adult STIs prevented under the program times the cost per STI case, where the latter is the weighted mean of serious cases (tertiary syphilis and HIV) and mild cases (primary and secondary syphilis).

We assumed equivalence in the intensity of existing versus new/expanded services, i.e., no enhancements other than the expansion of existing screening and treating activities. We calculated the cost of the prior maternal syphilis services as the cost of the new program times the ratio of the proportion tested and treated under the current level of coverage versus under the expanded program.

### Sensitivity analyses

In order to assess the influence of variations in the value of key model inputs on cost-effectiveness within each generic country scenario, we performed one-way sensitivity analyses on 20 variables. For the base case country scenarios, we defined a high prevalence of syphilis among pregnant women in ANC of 3%; this represents a plausible value based on empirical evidence from various countries. However, some published studies have reported prevalence estimates exceeding this value [27]. Therefore, we explored the effect of increasing the population prevalence of syphilis to 6% in sensitivity analysis. Additional variables explored related to the impact of the intervention included: the prevalence of active syphilis among pregnant women with a reactive syphilis serological test, test sensitivity of the RPR, current coverage of syphilis testing and treatment among ANC attendants, health service cost level (inpatient and outpatient), expected coverage of syphilis testing and treatment among ANC attendants in the intervention scenario, treatment performance, incidence of adverse pregnancy outcomes in untreated pregnancies, the expected number of adult syphilis cases averted for each syphilis positive pregnant woman treated, and the number of adult HIV infections averted per adult syphilis infection treated. We also included in the sensitivity analysis the cost of the syphilis serological test, the cost of a course of penicillin (where we assume a single dose instead of 3 doses of penicillin is used [28]), and all DALY estimates (i.e., the number of DALYs per infant infected with syphilis).

The ranges used for the sensitivity analysis are presented in Table 1. Results are displayed in modified spider diagrams showing effects on net costs and DALYs averted.

Finally, in order to test the combined effect of uncertainty in basic assumptions that were unrelated to the intervention, we performed four two-way sensitivity analyses. The inputs varied in these four analyses are: 1) DALYs associated with stillbirth, and adverse outcome incidence without treatment, 2) treatment performance, and adverse outcome incidence without treatment, 3) treatment performance, and adult syphilis averted per syphilis positive pregnancy treated, and 4) percentage of active syphilis among pregnant women with a reactive syphilis serological test, and adverse outcome incidence without treatment. We used the same ranges for these inputs as we used in the one-way sensitivity analyses.

## Results

### Base case

In the eight country scenarios, we estimate between 686 – 11,140 MTCT of syphilis AOs would be averted over four years with the expanded program, including 278 – 4,521 stillbirths, 124 – 2,012 neonatal deaths, 206 – 3,353 infected infants, and 77 – 1,255 premature or low birth weight infants. When factoring in adult syphilis and HIV cases that would be averted by treating cases of maternal syphilis, this translates to 5,754 – 93,484 DALYs that would be averted across the eight case scenarios.

Table 2 and Figure S1 present the cost, health impact, and cost-effectiveness findings for the base case values in the eight country settings. The estimated four-year cost of the expanded testing and treatment program ranges from $4,142,287 – $8,235,796 (2010 US dollars), assuming 1,000,000 pregnant women per year. When adjusted for offsetting savings due to averted AOs and replaced current services, net incremental costs range from net savings of $12,261,250 to a net cost of $1,736,807. Net savings are achieved in all four of the high prevalence country scenarios.

The MTCT of syphilis elimination program averts an estimated 5,754 – 93,484 DALYs over four years in the eight scenarios, yielding a cost-effectiveness ratio of $24 – $111 per DALY averted in the four scenarios with net costs. In the remaining four country scenarios, the program is cost saving, or dominant (more effective and less expensive as compared to the baseline); i.e., the intervention pays for itself in offset medical costs so no cost-effectiveness (CE) ratio needs to be calculated.

### Sensitivity analyses

**Figure 1:**
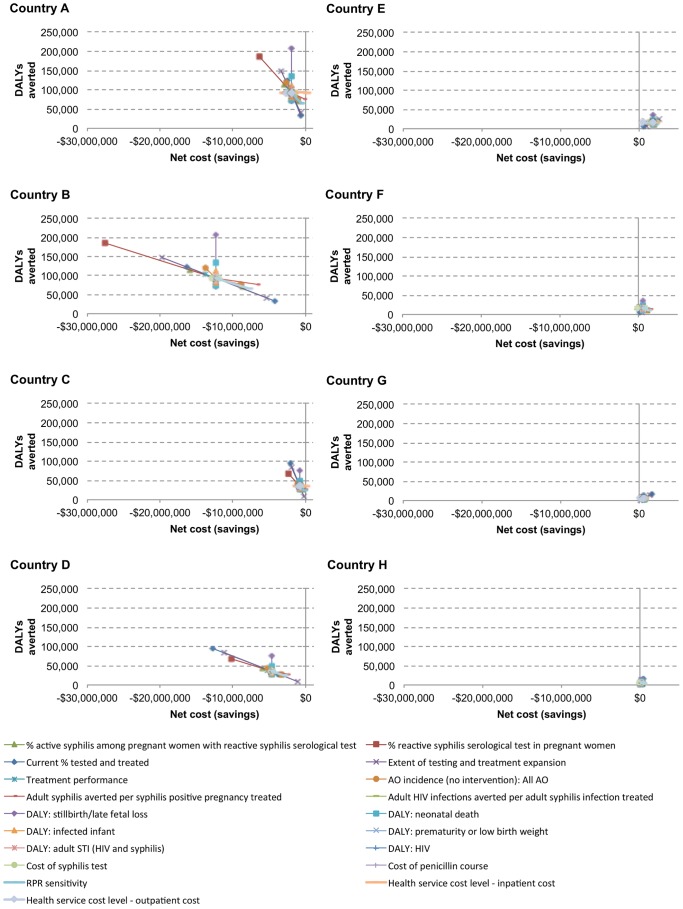
Sensitivity of net cost and DALYs averted to uncertainty in 20 key inputs. Costs are in 2010 USD. Eight country scenarios (A–H) are represented in panels. Scenarios A–D are high syphilis prevalence (3% in the base case), and scenarios E–H are low syphilis prevalence (0.5% in the base case). In scenarios A–D, the intervention remains cost saving across almost all sensitivity analysis values. In scenarios E–H, the intervention remains at least highly cost-effective across all input variations.

Results for one-way sensitivity analyses within scenarios are presented in Figure 1 (net cost and DALYs) and in Table S2 (net cost, DALYs, and CE ratios). Since the basic structure of the analysis presented in this article is a 3-way sensitivity analysis (three dichotomous factors generating 8 scenarios), these one-way factor variations test the robustness of each scenario.

In high syphilis prevalence scenarios (country scenarios A through D), the intervention remains cost saving across nearly all sensitivity analysis values; reducing the relative inpatient cost differential between South Africa and lower cost countries to 0.10 slightly worsens the cost-effectiveness ratio in scenario A (from cost-saving to $1/DALY averted). Increasing the background syphilis prevalence significantly increases the savings and benefits of the intervention. The net savings and DALYs averted move in tandem (essentially as a scale effect) according to the current coverage of testing and treatment, the coverage gain, and the risk of adverse events without treatment. Total DALYs averted, but not costs, are sharply affected by DALYs associated with stillbirth. Uncertainty in other factors has smaller effects.

In low prevalence scenarios (country scenarios E through H), the intervention remains highly cost-effective across all input variations. The intervention becomes cost saving in two country scenarios (F and H) when modifying two inputs: the prevalence of active syphilis among pregnant women with a reactive syphilis serological test (from 65% to 80%) and the cost of a syphilis serological test (from $2.30 to $1.82). Overall, however, there is much less absolute variation in costs and DALYs than in scenarios A through D (Figure 1). This is because the prevalence of syphilis is six times lower, so that proportionate changes in other factors (e.g. rise in coverage) modifies a much smaller base case result.

While lowering the inpatient cost ratio slightly worsens cost-effectiveness, lowering the outpatient cost ratio slightly improves cost-effectiveness. Lowering both the inpatient and outpatient cost level in tandem balances out the effect of each, yielding results more closely resembling the base case.

Switching from a three-dose course of penicillin to a single dose has a negligible effect on the cost-effectiveness of the expanded program (with the cost per DALY averted in the low prevalence scenarios changing from $24–$111 to $23–$111). This is understandable since the program is already cost saving in high prevalence scenarios, and only a small proportion of women require treatment in low prevalence scenarios.

The results are relatively sensitive to five main assumptions (detail in Table S2). The input with the greatest influence on cost-effectiveness is the sensitivity of the RPR test. If we assume that the RPR test has a sensitivity of 70.7%, the intervention remains cost saving in the four high-prevalence scenarios, and in the four low-prevalence scenarios the cost per DALY averted ranges from $103–$177 (compared to $24–$111 in the base case). Cost-effectiveness is also sensitive to the assumption of the horizontal spread of syphilis. If we assume that treatment of syphilis in pregnancy has a lesser effect on horizontal syphilis transmission than assumed in the base case (i.e., averts either 0.5 or 0 cases of adult syphilis instead of 1 case at baseline), the results are somewhat less favorable but remain qualitatively the same (see Text S1). Assuming zero cases of adult syphilis averted per case treated, the cost per DALY averted increases to $103–$157 for the intervention in the four low-prevalence scenarios (compared to $24–$111 in the base case). Cost-effectiveness is similarly sensitive to the risk of adverse events without treatment. The CE ratio is also very sensitive to the DALYs per stillbirth. Cost and DALY results are sensitive to scale factors such as the gain in coverage, but tend to rise and fall together, such that the CE ratio is relatively unaffected by these factors. Other factors, such as proportion of syphilis in pregnant women, have smaller magnitude effects on all outcomes.

Results for two-way sensitivity analyses within scenarios are presented in Table S3 (net cost, DALYs, and CE ratios). Varying the cases of adult syphilis averted per syphilis positive pregnancy treated (from 1 down to 0 cases) in tandem with the treatment performance (from 0.9 down to 0.7) yields the greatest effect on cost-effectiveness. In scenarios B and D, the intervention remains cost saving, though in scenarios A and C, the intervention changes from cost saving to carrying a cost per DALY averted of between $4–$6 per DALY averted. In the four low-prevalence scenarios, the cost per DALY averted increases to $133–$207 per DALY averted (compared to $24–$111 in the base case).

In the remaining two-way sensitivity analyses, varying the factors down to the minima in the ranges has no qualitative effect on the cost-effectiveness ratio in scenarios A through D (remains cost saving). In scenarios E through G, the cost-effectiveness ratio worsens slightly when the factors are lowered to their minima; however, varying the factors up to their maxima improves cost-effectiveness and the intervention becomes cost saving in scenarios F and H.

## Discussion

We found that eliminating congenital syphilis through an expanded screening and treatment program in antenatal care facilities would be cost saving in four of eight country scenarios examined. In the other four scenarios, the cost per DALY averted ranges from USD $24 – $111. According to the World Health Organization, an intervention is “cost-effective” if it costs up to three times the per-capita GDP of a country and is “highly cost-effective” if it costs less than the per capita GDP[29]. By WHO standards, therefore, the MTCT of syphilis elimination program can be considered highly cost-effective in all of the scenarios we explored, given that all countries have a per-capita GDP of at least $111 [30]. Although the cost-effectiveness results were calculated per one million pregnancies, the results are expected to scale, leaving the cost per DALY averted unchanged for countries of different sizes.

The four country scenarios in which the initiative would be cost saving are all high prevalence settings (i.e., prevalence of a reactive syphilis serological test in pregnancy of 3%). Though the cost of the program is roughly comparable to that in low prevalence ((0.5%) settings, high prevalence settings would be expected to achieve higher offsetting savings due to greater net averted disease costs. However, even in low prevalence settings, the program would be expected to avert approximately $300,000 – $2.6 million in medical costs from MTCT of syphilis and adult STI adverse outcomes. While the initiative is cost saving, or at least highly cost-effective, in all of the country scenarios examined, the most favorable findings (i.e., lowest net cost and highest number of DALYs averted) were observed in the scenario with a high maternal prevalence of test-positive syphilis, low level of current testing and treatment, and high service cost level. This is understandable considering countries that currently test and treat only a small proportion of ANC attendees would stand to benefit most from an expanded program. Moreover, countries in which the cost of medical services is high would be expected to pay more to treat AOs, and would see higher savings from averted disease costs in the MTCT of syphilis elimination program.

In addition to being cost-effective, integrating an expanded screening and treatment program into existing ANC and prevention of MTCT of HIV programs has been shown to be a feasible and efficient way to reduce the burden of MTCT of syphilis, and infant mortality and morbidity in general [4], [31]. Though attendance at ANC varies greatly, more than three-quarters of all pregnant women globally now receive at least one antenatal care visit [32], suggesting that ANC programs are widespread and generally well accepted. To enhance effectiveness, elimination programming should be coupled with a strong marketing/outreach component to promote early access to ANC, since this is the period of maximum effectiveness for maternal syphilis treatment [9]. An integrated program that focuses on early access to services would build on existing efforts to improve the quality of antenatal care, strengthening and maximizing the ability of these programs to improve maternal health and reduce infant mortality.

There are a few important limitations to this analysis. First, we did not model cost-effectiveness in specific countries, but rather in hypothetical scenarios with a pre-determined set of characteristics related to prevalence, care coverage, and health care services cost (see Table S1 for countries resembling the eight hypothetical scenarios). Given the varied priorities and resource constraints in many countries, it will be important for health care entities to assess the cost-effectiveness of expanded syphilis screening and treatment within existing ANC services under their specific set of country characteristics. One way to do this is to adapt modeling techniques, such as those used in this study, to the context of individual countries through the use of national data and targets [33]. Nonetheless, by modeling cost-effectiveness in eight different scenarios, this analysis presents findings that are likely robust over a wide range of settings.

Second, when quantifying the cost of AOs, we included only the direct medical costs of care and treatment. We did not include indirect costs, such as lost productivity, and special educational needs of infants with syphilis infection, since such costs are very difficult to quantify. However, including these costs would have made the cost-effectiveness estimates of the initiative even more favorable. Third, given the range of syphilis serological tests and the absence of quantitative or confirmatory tests in most low-income countries, we made a simplifying assumption that 65% of positive serologic tests reported in surveillance were true positives. This was based on two types of data from studies in low-income settings: the positive predictive value of a rapid test against a gold standard of laboratory based RPR and VDRL [17], [18]; and the proportion of RPR positive tests with a titer of at least 1:8 [19]. This titer suggests sufficient treponemal load for vertical transmission and excludes most serofast or biologic false positive tests. Future analyses would benefit from explicit modeling of test sensitivity and specificity. Fourth, we assumed treatment with penicillin would be based on a reactive serological test, and we did not attempt to estimate the health impact of any potential side effects of treatment for women who are biologically false positive. However, penicillin has no known negative impact on pregnancy in women without penicillin allergy, and common side effects such as diarrhea have small DALY effects when weighed against the benefits of prompt treatment before a confirmatory test.

Finally, the value of some of the inputs was uncertain due to a scarcity of data in the literature (i.e., the cost of primary syphilis). Where data were lacking or imperfect, we made adjustments or assumptions for base case values, and performed multiple one-way sensitivity analyses and four two-way sensitivity analyses over a range of input values. For example, in the base case, we assumed a DALY benefit from treatment due to reduced AOs in the infant and reduced STIs in adults. Since the benefits to the index patient and others in terms of reduced adult STIs are uncertain, we performed sensitivity analysis to explore the effect of a lesser influence of treatment on horizontal transmission. Even assuming no DALY benefits in adults from treatment, the results remain qualitatively similar. Indeed, the MTCT of syphilis elimination program remained cost saving or highly cost-effective across all of the inputs explored.

Our analysis suggests that integrating expanded syphilis screening and treatment into ANC programs would be cost saving or highly cost-effective in all scenarios examined. Countries with high maternal syphilis prevalence, low current service coverage, and high healthcare cost would likely benefit most. Future analyses can be tailored to specific countries using local epidemiologic and programmatic data. Based on realistic assessments of human resource and infrastructure capacity, specific targets for screening and treatment within existing ANC services will be needed to assess progress on a country level. Country-specific targets for impact indicators, such as the rate of stillbirths and the rate of clinical syphilis, will also be needed to assess the program's progress towards the goal of elimination of MTCT of syphilis.

## Supporting Information

Figure S1 **Intervention cost and net incremental cost of scaled-up screening and treatment for MTCT syphilis prevention.** Costs are in 2010 USD. Intervention cost and net incremental cost (savings) are presented for country scenarios A–H. Scenario factors on the horizontal axis: Prevalence of syphilis by serological testing (high prevalence  = 3%; low prevalence  = 0.5%); Coverage of current syphilis screening and treatment in ANC (high coverage  = 70%; low coverage  = 20%); Cost of health services (i.e., health care cost structure, including the cost of MTCT of syphilis AOs; high cost  = 1; low cost  = 0.25 based on WHO CHOICE data (http://www.who.int/choice/en/). Implementing expanded testing and treatment of syphilis in ANC generates net savings in settings with high maternal syphilis prevalence (3%), especially in scenarios where the cost of care and treatment is high. In settings with low maternal syphilis prevalence (0.5%), the intervention yields net costs of ∼$140,000 – $1.7 million. Net costs are substantially lower than intervention costs due to the offsetting savings resulting from averted MTCT of syphilis adverse outcomes and adult syphilis and HIV, as well as prior syphilis testing and treatment services replaced by the expanded program.
(TIF)

Table S1 **Countries resembling the eight generic case scenarios.**
(DOCX)

Table S2 **One-way sensitivity analysis findings (net costs, DALYs averted, and CE ratios) for eight country scenarios and 20 key inputs.**
(DOCX)

Table S3 **Two-way sensitivity analysis findings (net costs, DALYs averted, and CE ratios) for eight country scenarios and key inputs.**
(DOCX)

Text S1 **Calculation of DALYs averted by preventing the horizontal spread of adult syphilis and by preventing syphilis-attributable HIV cases.**
(DOCX)
