# Supplementary figures and images for: The Cost and Cost-Effectiveness of Scaling up Screening and Treatment of Syphilis in Pregnancy: A Model
Source: PLoS One. 2014 Jan 29;9(1):e87510. doi: 10.1371/journal.pone.0087510 (PMC3906198; doi:10.1371/journal.pone.0087510)

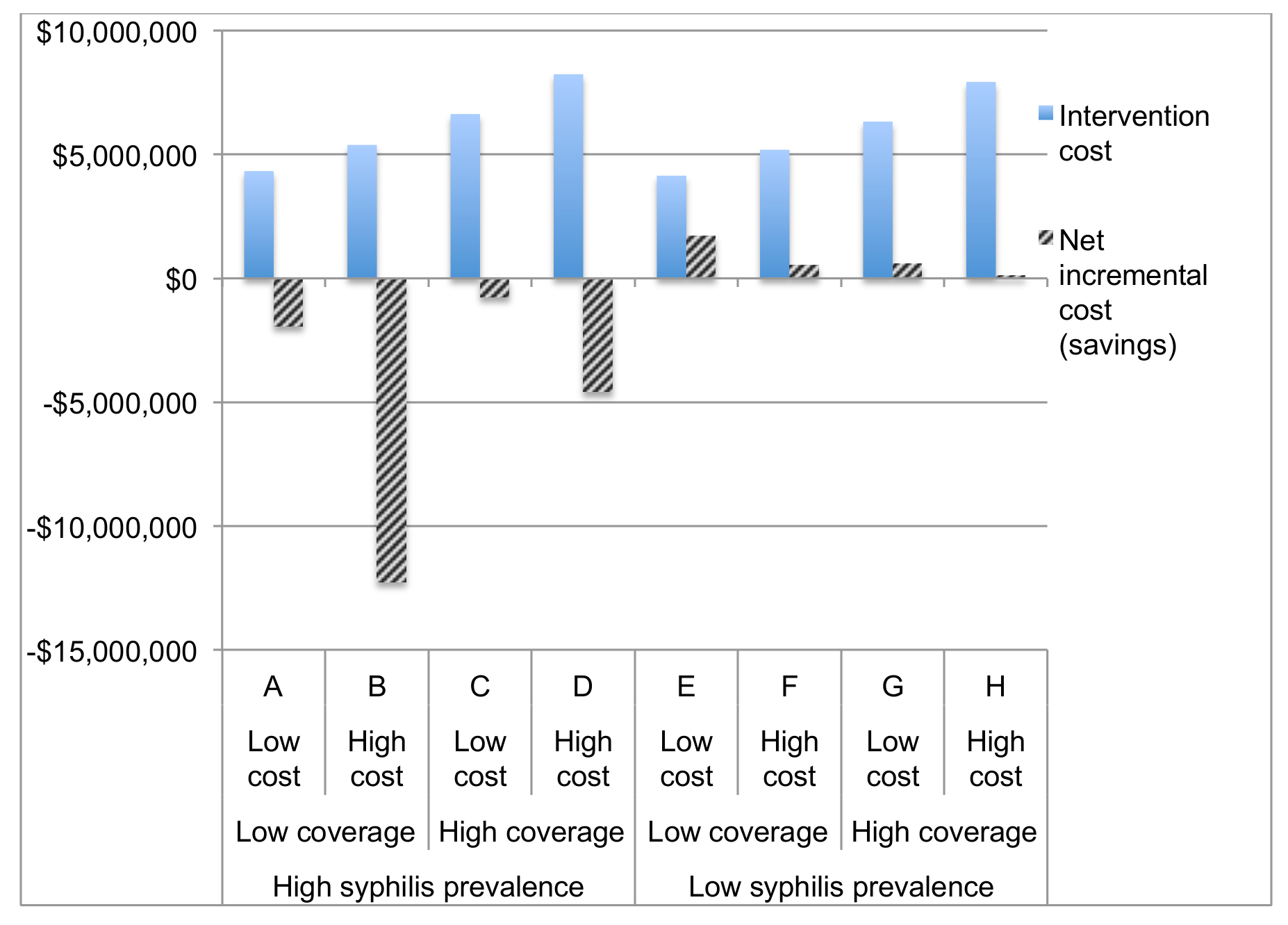

Supplement: Figure S1 — Intervention cost and net incremental cost of scaled-up screening and treatment for MTCT syphilis prevention. Costs are in 2010 USD. Intervention cost and net incremental cost (savings) are presented for country scenarios A–H. Scenario factors on the horizontal axis: Prevalence of syphilis by serological testing (high prevalence = 3%; low prevalence = 0.5%); Coverage of current syphilis screening and treatment in ANC (high coverage = 70%; low coverage = 20%); Cost of health services (i.e., health care cost structure, including the cost of MTCT of syphilis AOs; high cost = 1; low cost = 0.25 based on WHO CHOICE data (http://www.who.int/choice/en/). Implementing expanded testing and treatment of syphilis in ANC generates net savings in settings with high maternal syphilis prevalence (3%), especially in scenarios where the cost of care and treatment is high. In settings with low maternal syphilis prevalence (0.5%), the intervention yields net costs of ∼$140,000 – $1.7 million. Net costs are substantially lower than intervention costs due to the offsetting savings resulting from averted MTCT of syphilis adverse outcomes and adult syphilis and HIV, as well as prior syphilis testing and treatment services replaced by the expanded program. (TIF) [file pone.0087510.s001.tif]
