# Supplementary material for: The Cost and Cost-Effectiveness of Scaling up Screening and Treatment of Syphilis in Pregnancy: A Model
Source: PLoS One. 2014 Jan 29;9(1):e87510. doi: 10.1371/journal.pone.0087510 (PMC3906198; doi:10.1371/journal.pone.0087510)
Supplement: Table S1 — Countries resembling the eight generic case scenarios. (DOCX) [file pone.0087510.s002.docx]

Table S1 – Countries resembling the eight generic case scenarios

|  | **Case Scenario** | | | | | | | |
| --- | --- | --- | --- | --- | --- | --- | --- | --- |
|  | **A** | **B** | **C** | **D** | **E** | **F** | **G** | **H** |
| Syphilis prevalence/ current coverage/ cost level | hi / lo / lo | hi / lo / hi | hi / hi / lo | hi / hi / hi | lo / lo / lo | lo / lo / hi | lo / hi / lo | lo / hi / hi |
|  | 0.03 / 0.2 / 0.25 | 0.03 / 0.2 / 1 | 0.03 / 0.7 / 0.25 | 0.03 / 0.7 / 1 | 0.005 / 0.2 / 0.25 | 0.005 / 0.2 / 1 | 0.005 / 0.7 / 0.25 | 0.005 / 0.7 / 1 |
| **Countries** | Tanzania, Madagascar, Zambia | Equatorial Guinea, Swaziland, Indonesia | Mozambique, Haiti | South Africa, Namibia, Paraguay, Grenada, Chile | Nicaragua, Myanmar | Guatemala, Iraq | Cote d'Ivoire, Guyana, India | Mauritius, Cape Verde, Barbados, Maldives |
|  |  |  |  |  |  |  |  |  |
|  |  |  |  |  |  |  |  |  |

The eight generic case scenarios described in our analysis were defined by three contextual factors predictive of the incremental cost and impact of scaled-up syphilis screening and treatment. These factors are 1) the prevalence of syphilis among pregnant women attending ANC, 2) the current level of syphilis testing and treatment coverage in ANC, and 3) the relative cost of health services.

Using publicly available data on ANC syphilis prevalence [[1](#_ENREF_1)], ANC syphilis testing [[2](#_ENREF_2)], and health care service costs [[3](#_ENREF_3)], we created Table S1 to highlight examples of countries that resemble each of the eight scenarios. ANC syphilis testing was used since publicly available data on ANC syphilis testing and treatment were not available. Countries listed in Table S1 have values that match or approximate the base case assumptions for each of the three case-defining factors.

We defined the cost of health services as the relative cost of comparable health services based on WHO CHOICE unit cost data. For inpatient care, we specified the relative unit cost differential between the country for which we obtained cost data (South Africa) and lower cost countries as 1 to 0.25. For outpatient care, the analogous ratio in WHO CHOICE is 1 to 0.75. The cost of a secondary level hospital inpatient visit per bed day (2008) = USD $57.65 [[3](#_ENREF_3)]. In Table S1, countries were classified as low cost if their cost of a secondary level inpatient visit per bed day was 25% or less of South Africa's, and high cost if their cost was more than 25% of South Africa's.

**References**

1. World Health Organization (2008-2011) Global Health Observatory Data Repository. Antenatal care attendees who were positive for syphilis by country.

2. World Health Organization (2008-2011) Global Health Observatory Data Repository. Antenatal care (ANC) attendees tested for syphilis at first ANC visit by country.

3. World Health Organization (2013) WHO-CHOICE. Country-specific unit costs (2008).
