# Supplementary material for: The Cost and Cost-Effectiveness of Scaling up Screening and Treatment of Syphilis in Pregnancy: A Model
Source: PLoS One. 2014 Jan 29;9(1):e87510. doi: 10.1371/journal.pone.0087510 (PMC3906198; doi:10.1371/journal.pone.0087510)
Supplement: Table S2 — One-way sensitivity analysis findings (net costs, DALYs averted, and CE ratios) for eight country scenarios and 20 key inputs. (DOCX) [file pone.0087510.s003.docx]

Table S2 – One-way sensitivity analysis findings (net costs, DALYs averted, and CE ratios) for eight country scenarios and 20 key inputs.

|  |  | A | B | C | D | E | F | G | H |
| --- | --- | --- | --- | --- | --- | --- | --- | --- | --- |
| Base case | Total DALYs averted | 93,484 | 93,484 | 34,518 | 34,518 | 15,584 | 15,584 | 5,754 | 5,754 |
|  | Net costs | -$1,943,017 | -$12,261,250 | -$765,563 | -$4,587,778 | $1,736,807 | $543,472 | $593,188 | $140,282 |
|  | ICER (CS) | n/a | n/a | n/a | n/a | $935 | $293 | $865 | $205 |
|  | ICER (DALY) | n/a | n/a | n/a | n/a | $111 | $35 | $103 | $24 |
| *Variable* |  |  |  |  |  |  |  |  |  |
| % active syphilis among pregnant women with reactive syphilis (0.65; 0.5,0.8) | Total DALYs averted | 71,911; 115,057 | 71,911; 115,057 | 26,553; 42,484 | 26,553; 42,484 | 11,988; 19,180 | 11,988; 19,180 | 4,426; 7,082 | 4,426; 7,082 |
|  | Net costs | -$891,957;  -$2,994,078 | -$8,682,663; -$15,839,837 | -$377,466;  -$1,153,660 | -$3,266,408; -$5,909,147 | $1,912,588; $1,561,027 | $1,140,507;  -$53,563 | $658,094; $528,282 | $360,733;  -$80,170 |
|  | ICER (CS) | n/a | n/a | n/a | n/a | $1,339; $683 | $799; n/a | $1,248; $626 | $684; n/a |
|  | ICER (DALY) | n/a | n/a | n/a | n/a | $160; $81 | $95; n/a | $149; $75 | $81; n/a |
| % reactive syphilis serological test in pregnant women (0.03; 0.03,0.6) | Total DALYs averted | 93,484; 186,933 | 93,484; 186,933 | 34,518; 69,024 | 34,518; 69,024 | - | - | - | - |
|  | Net costs | -$1,943,017; -$6,333,417 | -$12,261,250; -$27,601,526 | -$765,563;  -$2,386,689 | -$4,587,778; -$10,252,074 | - | - | - | - |
|  | ICER (CS) | n/a | n/a | n/a | n/a | - | - | - | - |
|  | ICER (DALY) | n/a | n/a | n/a | n/a | - | - | - | - |
| Test sensitivity of RPR (100; 70.7,100) | Total DALYs averted | 66,093; 93,484 | 66,093; 93,484 | 24,404; 34,518 | 24,404; 34,518 | 11,018; 15,584 | 11,018; 15,584 | 4,068; 5,754 | 4,068; 5,754 |
|  | Net costs | -$648,271;  -$1,943,017 | -$7,758,190; -$12,261,250 | -$287,486;  -$765,563 | -$2,925,052;  -$4,587,778 | $1,953,365; $1,736,807 | $1,294,748; $543,472 | $673,150; $593,188 | $417,686; $140,282 |
|  | ICER (CS) | n/a | n/a | n/a | n/a | $1,488; $935 | $986; $293 | $1,389; $865 | $862; $205 |
|  | ICER (DALY) | n/a | n/a | n/a | n/a | $177; $111 | $118; $35 | $165; $103 | $103; $24 |
| Current proportion tested and treated (low coverage = 0.2; 0.1,0.4; high coverage = 0.7; 0.5,0.72) | Total DALYs averted | 124,235; 31,981 | 124,235; 31,981 | 96,021; 28,368 | 96,021; 28,368 | 20,710; 5,331 | 20,710; 5,331 | 16,007; 4,729 | 16,007; 4,729 |
|  | Net costs | -$2,582,168; -$664,716 | -$16,294,556  ; -$4,194,638 | -$2,129,595; -$629,160 | -$12,761,992; -$3,770,356 | $2,308,125; $594,171 | $722,246; $185,925 | $1,650,093; $487,498 | $390,227; $115,287 |
|  | ICER (CS) | n/a | n/a | n/a | n/a | $935; $935 | $293; $293 | $865; $865 | $205; $205 |
|  | ICER (DALY) | n/a | n/a | n/a | n/a | $111; $111 | $35; $35 | $103; $103 | $24; $24 |
| Health service cost level – inpatient∫ (low cost scenarios only = 0.25; 0.10,0.33) | Total DALYs averted | 93,484; 93,484 | - | 34,518; 34,518 | - | 15,584; 15,584 | - | 5,754; 5,754 | - |
|  | Net costs | $120,629;  - $3,043,629 | - | -$1,120;  -$1,173,266 | - | $1,975,474; $1,609,518 | - | $683,769; $544,878 | - |
|  | ICER (CS) | 11; n/a | - | n/a | - | $1,064; $867 | - | $997; $795 | - |
|  | ICER (DALY) | 1; n/a | - | n/a | - | $127; $103 | - | $119; $95 | - |
| Health service cost level – outpatient (low cost scenarios only = 0.75; 0.20,0.75) | Total DALYs averted | 93,484; 93,484 | - | 34,518; 34,518 | - | 15,584; 15,584 | - | 5,754; 5,754 | - |
|  | Net costs | -$2,681,997; -$1,943,017 | - | -$1,011,421; -$765,563 | - | $455,629; $1,736,807 | - | $147,127; $593,188 | - |
|  | ICER (CS) | n/a | - | n/a | - | $245; $935 | - | $215; $865 | - |
|  | ICER (DALY) | n/a | - | n/a | - | $29; $111 | - | $26; $103 | - |
| Health service cost level – combined inpt. and outpt. (low cost scenarios only) | Total DALYs averted | 93,484; 93,484 | - | 34,518; 34,518 | - | 15,584; 15,584 | - | 5,754; 5,754 | - |
|  | Net costs | -$551,171;  -$3,079,458 | - | -$224,627;  -$1,185,186 | - | $810,767; $1,547,400 | - | $278,259; $523,251 | - |
|  | ICER (CS) | n/a | - | n/a | - | $437; $833 | - | $406; $763 | - |
|  | ICER (DALY) | n/a | - | n/a | - | $52, $99 | - | $48; $91 | - |
| Extent of testing and treatment expansion (intervention) (low coverage = 0.504; 0.33,0.684; high coverage = 0.812; 0.727,0.970) | Total DALYs averted | 41,822; 148,836 | 41,822; 148,836 | 8,226; 83,120 | 8,226; 83,120 | 6,972; 24,811 | 6,972; 24,811 | 1,371; 13,856 | 1,371; 13,856 |
|  | Net costs | -$730,789;  -$3,300,957 | -$5,311,518; -$19,781,598 | -$182,439;  -$1,927,748 | -$1,093,301; -$11,153,173 | $915,448; $2,557,711 | $416,910; $604,867 | $141,361; $134,137 | $33,430; $232,037 |
|  | ICER (CS) | n/a | n/a | n/a | n/a | $1,102; $865 | $502; $205 | $865; $814 | $205; $141 |
|  | ICER (DALY) | n/a | n/a | n/a | n/a | $131; $106 | $60; $24 | $103; $97 | $24; $17 |
| Treatment performance (0.9; 0.7,0.99) | Total DALYs averted | 72,710; 102,832 | 72,710; 102,832 | 26,848; 37,970 | 26,848; 37,970 | 12,121; 17,142 | 12,121; 17,142 | 4,476; 6,330 | 4,476; 6,330 |
|  | Net costs | -$930,885;  -$2,398,477 | -$8,815,204; -$13,811,971 | -$391,840;  -$933,738 | -$3,315,348; -$5,160,371 | $1,906,077; $1,660,636 | $1,118,394; $284,757 | $655,690; $565,062 | $352,568; $44,753 |
|  | ICER (CS) | n/a | n/a | n/a | n/a | $1,320; $813 | $774; $139 | $1,230; $749 | $661; $59 |
|  | ICER (DALY) | n/a | n/a | n/a | n/a | $157; $97 | $92; $17 | $147; $89 | $79; $7 |
| AO incidence (no intervention): All AO (0.52; 0.4,0.7) | Total DALYs averted | 75,699; 120,322 | 75,699; 120,322 | 27,951; 44,428 | 27,951; 44,428 | 12,620; 20,057 | 12,620; 20,057 | 4,660; 7,406 | 4,660; 7,406 |
|  | Net costs | -$1,108,661; -$2,647,483 | -$8,884,299; -$13,779,200 | -$457,482; -$1,025,682 | -$3,340,861; -$5,148,271 | $1,875,901; $1,619,431 | $1,106,332; $290,515 | $644,548; $549,848 | $348,114; $46,879 |
|  | ICER (CS) | n/a | n/a | n/a | n/a | $1,319; $650 | $778; $117 | $1,227; $598 | $663; $51 |
|  | ICER (DALY) | n/a | n/a | n/a | n/a | $149; $81 | $88; $14 | $138; $74 | $75; $6 |
| Adult syphilis averted per syphilis positive pregnancy treated (1.0; 0.0,1.0) | Total DALYs averted | 77,062; 93,484 | 77,062; 93,484 | 28,455; 34,518 | 28,455; 34,518 | 12,844; 15,584 | 12,844; 15,584 | 4,742; 5,754 | 4,742; 5,754 |
|  | Net costs | -$251,861;  -$1,943,017 | -$6,581,361; -$12,261,250 | -$141,115;  -$765,563 | -$2,490,516;  -$4,587,778 | $2,021,282; $1,736,807 | $1,492,736; $543,472 | $698,229; $593,188 | $490,791; $140,282 |
|  | ICER (CS) | n/a | n/a | n/a | n/a | $1,089; $935 | $804; $293 | $1,018; $865 | $716; $205 |
|  | ICER (DALY) | n/a | n/a | n/a | n/a | $157; $111 | $116; $35 | $147; $103 | $103; $24 |
| Adult HIV infections averted per adult syphilis infection averted (0.001; 0.000,0.001) | Total DALYs averted | 93,352; 93,484 | 93,352; 93,484 | 34,470; 34,518 | 34,470; 34,518 | 15,559; 15,584 | 15,559; 15,584 | 5,745; 5,754 | 5,745; 5,754 |
|  | Net costs | -$1,835,241; -$1,943,017 | -$12,153,473; -$12,261,250 | -$725,767;  -$765,563 | -$4,547,982; -$4,587,778 | $1,757,386; $1,736,807 | $564,051; $543,472 | $600,787; $593,188 | $147,880; $140,282 |
|  | ICER (CS) | n/a | n/a | n/a | n/a | $947; $935 | $304; $293 | $876; $865 | $216; $205 |
|  | ICER (DALY) | n/a | n/a | n/a | n/a | $113; $111 | $36; $35 | $105; $103 | $26; $24 |
| DALY: stillbirth/late fetal loss (4.95; 0.00,30.00) | Total DALYs averted | 71,106; 206,731 | 71,106; 206,731 | 26,255; 76,334 | 26,255; 76,334 | 11,854; 34,458 | 11,854; 34,458 | 4,377; 12,724 | 4,377; 12,724 |
|  | Net costs | -$1,943,017; -$1,943,017 | -$12,261,250;  -$12,261,250 | -$765,563;  -$765,563 | -$4,587,778; -$4,587,778 | $1,736,807; $1,736,807 | $543,472; $543,472 | $593,188; $593,188 | $140,282; $140,282 |
|  | ICER (CS) | n/a | n/a | n/a | n/a | $935; $935 | $293; $293 | $865; $865 | $205; $205 |
|  | ICER (DALY) | n/a | n/a | n/a | n/a | $147; $50 | $46; $16 | $136; $47 | $32; $11 |
| DALY: neonatal death (9.40; 0.00,30.00) | Total DALYs averted | 74,574; 134,924 | 74,574; 134,924 | 27,536; 49,820 | 27,536; 49,820 | 12,432; 22,491 | 12,432; 22,491 | 4,591; 8,305 | 4,591; 8,305 |
|  | Net costs | -$1,943,017; -$1,943,017 | -$12,261,250;  -$12,261,250 | -$765,563;  -$765,563 | -$4,587,778; -$4,587,778 | $1,736,807; $1,736,807 | $543,472; $543,472 | $593,188; $593,188 | $140,282; $140,282 |
|  | ICER (CS) | n/a | n/a | n/a | n/a | $935; $935 | $293; $293 | $865; $865 | $205; $205 |
|  | ICER (DALY) | n/a | n/a | n/a | n/a | $140; $77 | $44; $24 | $129; $71 | $31; $17 |
| DALY: infected infant (9.48; 6.00,15.00) | Total DALYs averted | 81,816; 111,991 | 81,816; 111,991 | 30,210; 41,352 | 30,210; 41,352 | 13,639; 18,668 | 13,639; 18,668 | 5,036; 6,893 | 5,036; 6,893 |
|  | Net costs | -$1,943,017; -$1,943,017 | -$12,261,250;  -$12,261,250 | -$765,563;  -$765,563 | -$4,587,778; -$4,587,778 | $1,736,807; $1,736,807 | $543,472; $543,472 | $593,188; $593,188 | $140,282; $140,282 |
|  | ICER (CS) | n/a | n/a | n/a | n/a | $935; $935 | $293; $293 | $865; $865 | $205; $205 |
|  | ICER (DALY) | n/a | n/a | n/a | n/a | $127; $93 | $40; $29 | $118; $86 | $28; $20 |
| DALY: prematurity or low birth weight (3.18; 1.59,4.77) | Total DALYs averted | 91,489; 95,479 | 91,489; 95,479 | 33,782; 35,255 | 33,782; 35,255 | 15,251; 15,916 | 15,251; 15,916 | 5,631; 5,877 | 5,631; 5,877 |
|  | Net costs | -$1,943,017; -$1,943,017 | -$12,261,250;  -$12,261,250 | -$765,563;  -$765,563 | -$4,587,778; -$4,587,778 | $1,736,807; $1,736,807 | $543,472; $543,472 | $593,188; $593,188 | $140,282; $140,282 |
|  | ICER (CS) | n/a | n/a | n/a | n/a | $935; $935 | $293; $293 | $865; $865 | $205; $205 |
|  | ICER (DALY) | n/a | n/a | n/a | n/a | $114; $109 | $36; $34 | $105; $101 | $25; $24 |
| DALY: adult STI (HIV and syphilis) (0.759; 0.380,1.139) | Total DALYs averted | 85,288; 101,718 | 85,288; 101,718 | 31,492; 37,559 | 31,492; 37,559 | 14,215; 16,954 | 14,215; 16,954 | 5,249; 6,260 | 5,249; 6,260 |
|  | Net costs | -$1,943,017; -$1,943,017 | -$12,261,250;  -$12,261,250 | -$765,563;  -$765,563 | -$4,587,778; -$4,587,778 | $1,736,807; $1,736,807 | $543,472; $543,472 | $593,188; $593,188 | $140,282; $140,282 |
|  | ICER (CS) | n/a | n/a | n/a | n/a | $935; 935 | $293; $293 | $865; $865 | $205; $205 |
|  | ICER (DALY) | n/a | n/a | n/a | n/a | $122; 102 | $38; $32 | $113; $95 | $27; $22 |
| DALY: HIV (7.20; 3.60,7.20) | Total DALYs averted | 93,424; 93,484 | 93,424; 93,484 | 34,496; 34,518 | 34,496; 34,518 | $15,572; $15,584 | 15,572; 15,584 | 5,750; 5,754 | 5,750; 5,754 |
|  | Net costs | -$1,943,017; -$1,943,017 | -$12,261,250;  -$12,261,250 | -$765,563;  -$765,563 | -$4,587,778; -$4,587,778 | $1,736,807; $1,736,807 | $543,472; $543,472 | $593,188; $593,188 | $140,282; $140,282 |
|  | ICER (CS) | n/a | n/a | n/a | n/a | $935; $935 | $293; $293 | $865; $865 | $205; $205 |
|  | ICER (DALY) | n/a | n/a | n/a | n/a | $112; $111 | $35; $35 | $103; $103 | $24; $24 |
| Cost of syphilis serological test (low cost = 1.83; 1.48,2.22; high cost = 2.30; 1.82,2.56)) | Total DALYs averted | 93,484; 93,484 | 93,484; 93,484 | 34,518; 34,518 | 34,518; 34,518 | $15,584; $15,584 | 15,584; 15,584 | 5,754; 5,754 | 5,754; 5,754 |
|  | Net costs | -$2,419,284; -$1,419,462 | -$12,909,783;  -$11,909,961 | -$932,166;  -$582,418 | -$4,814,641; -$4,464,894 | $1,260,540; $2,260,363 | -$105,061; $894,761 | $426,585; $776,333 | -$86,582; $263,166 |
|  | ICER (CS) | n/a | n/a | n/a | n/a | $679; $1,217 | n/a; $482 | $622; $1,132 | n/a; $384 |
|  | ICER (DALY) | n/a | n/a | n/a | n/a | $81; $145 | n/a; $57 | $74; $135 | n/a; $46 |
| Cost of penicillin course (low cost = 3.72; 1.39,372; high cost = 3.79; 1.46,3.79) | Total DALYs averted | 93,484; 93,484 | 93,484; 93,484 | 34,518; 34,518 | 34,518; 34,518 | 15,584; 15,584 | 15,584; 15,584 | 5,754; 5,754 | 5,754; 5,754 |
|  | Net costs | -$2,027,990; -$1,943,017 | -$12,346,223; -$12,261,250 | -$796,939;  -$765,563 | -$4,619,153; -$4,587,778 | $1,722,645; $1,736,807 | $529,310; $543,472 | $587,959; $593,188 | $135,052; $140,282 |
|  | ICER (CS) | n/a | n/a | n/a | n/a | $928; $935 | $285; $293 | $858; $865 | $197; $205 |
|  | ICER (DALY) | n/a | n/a | n/a | n/a | $111; $111 | $34; $35 | $102; $103 | $23; $24 |

* Table shows lower and upper bound values for total DALYs averted, net costs, ICER (CS) and ICER (DALY) for each one-way sensitivity analysis and for each of the eight country scenarios. ICER (CS) is the incremental cost-effectiveness ratio expressed as cost per congenital syphilis (CS) case averted. Base case and minimum and maximum values for each variable are presented in the table after the variable name as follows, “variable (base case; minimum, maximum).” See Table 1 for detailed base case inputs and assumptions for one-way sensitivity analyses.
