# Supplementary material for: The Cost and Cost-Effectiveness of Scaling up Screening and Treatment of Syphilis in Pregnancy: A Model
Source: PLoS One. 2014 Jan 29;9(1):e87510. doi: 10.1371/journal.pone.0087510 (PMC3906198; doi:10.1371/journal.pone.0087510)
Supplement: Table S3 — Two-way sensitivity analysis findings (net costs, DALYs averted, and CE ratios) for eight country scenarios and key inputs. (DOCX) [file pone.0087510.s004.docx]

Table S3 – Two-way sensitivity analysis findings (net costs, DALYs averted, and CE ratios) for eight country scenarios and key inputs.

|  |  |  | A | B | C | D | E | F | G | H |
| --- | --- | --- | --- | --- | --- | --- | --- | --- | --- | --- |
| Base case (b.c.) |  | Total DALYs averted | 93,484 | 93,484 | 34,518 | 34,518 | 15,584 | 15,584 | 5,754 | 5,754 |
|  |  | Net costs | -$1,943,017 | -$12,261,250 | -$765,563 | -$4,587,778 | $1,736,807 | $543,472 | $593,188 | $140,282 |
|  |  | ICER (CS) | n/a | n/a | n/a | n/a | $935 | $293 | $865 | $205 |
|  |  | ICER (DALY) | n/a | n/a | n/a | n/a | $111 | $35 | $103 | $24 |
| Variables | **Justification** |  |  |  |  |  |  |  |  |  |
| DALYs assoc. with stillbirth AND AO incidence without treatment | Cost-effectiveness relatively sensitive to both inputs | Total DALYs averted | 58,105; 271,406 | 58,105; 271,406 | 21,455; 100,215 | 21,455; 100,215 | 9,687; 45,237 | 9,687; 45,237 | 3,577; 16,704 | 3,577; 16,704 |
|  |  | Net costs | -$1,251,106; -$2,896,761 | -$9,888,276; -$15,536,161 | -$510,079; -$1,117,726 | -$3,711,573; -$5,797,017 | $1,852,161; $1,577,885 | $939,003;  -$2,311 | $635,782; $534,507 | $286,329;  -$61,245 |
|  |  | ICER (CS) | n/a | n/a | n/a | n/a | $1,302; $634 | $660; n/a | $1,210; $581 | $545; n/a |
|  |  | ICER (DALY) | n/a | n/a | n/a | n/a | $191; $35 | $97; n/a | $178; $32 | $80; n/a |
| Treatment performance AND AO incidence without treatment | If treatment not as effective as in b.c., might not be CE if AO incidence without treatment is lower than assumed in b.c. | Total DALYs averted | 58,530; 131,496 | 58,530; 131,496 | 21,612; 48,554 | 21,612; 48,554 | 9,757; 21,919 | 9,757; 21,919 | 3,603; 8,094 | 3,603; 8,094 |
|  |  | Net costs | -$392,732;  -$3,447,595 | -$6,969,557;  -$17,414,374 | -$193,130;  -$1,321,118 | -$2,633,855; -$6,490,535 | $1,995,797; $1,485,821 | $1,426,029;  -$315,605 | $688,818; $500,513 | $466,160;  - $176,927 |
|  |  | ICER (CS) | n/a | n/a | n/a | n/a | $1,804; $543 | $1,289; n/a | $1,686; $495 | $1,141; n/a |
|  |  | ICER (DALY) | n/a | n/a | n/a | n/a | $205; $68 | $146; n/a | $191; $62 | $129; n/a |
| Adult syphilis averted per pregnant case treated AND Treatment performance | CE sensitive to cases of adult syphilis averted per pregnancy treated. If fewer cases averted and lower treatment performance, CE might be reduced considerably | Total DALYs averted | 59,937; 84,768 | 59,937; 84,768 | 22,131; 31,300 | 22,131; 31,300 | 9,990; 14,128 | 9,990; 14,128 | 3,689; 5,217 | 3,689; 5,217 |
|  |  | Net costs | $384,459;  -$538,205 | -$4,397,512;  -$7,564,093 | $93,842;  -$246,845 | -$1,684,144;  -$2,853,384 | $2,127,336; $1,973,558 | $1,856,711; $1,328,947 | $737,388 $680,607 | $625,186; $430,313 |
|  |  | ICER (CS) | n/a | n/a | n/a | n/a | $1,473; $966 | $1,286; $651 | $1,383; $903 | $1,172; $571 |
|  |  | ICER (DALY) | n/a | n/a | n/a | n/a | $213; $140 | $186; $94 | $200; $130 | $169; $82 |
| % active syphilis among reactive cases AND AO incidence without treatment | To show uncertainty in basic assumptions that are not related to the intervention | Total DALYs averted | 57,887; 147,128 | 57,887; 147,128 | 21,374; 54,326 | 21,374; 54,326 | 9,650; 24,525 | 9,650; 24,525 | 3,563; 9,056 | 3,563; 9,056 |
|  |  | Net costs | -$359,717;  - $4,167,916 | -$6,857,299; -$19,870,497 | -$180,940;  -$1,587,092 | -$2,592,404;  -$7,397,442 | $2,001,321;  $1,365,430 | $1,444,761;  -$725,296 | $690,858; $456,060 | $473,077;  -$328,203 |
|  |  | ICER (CS) | n/a | n/a | n/a | n/a | $1,829; $446 | $1,320; n/a | $403; $1,171 | $1,171; n/a |
|  |  | ICER (DALY) | n/a | n/a | n/a | n/a | $207; $56 | $150; n/a | $50, $133 | $133; n/a |

* Table shows lower and upper bound values for total DALYs averted, net costs, ICER (CS) and ICER (DALY) for each two-way sensitivity analysis and for each of the eight country scenarios. ICER (CS) is the incremental cost-effectiveness ratio expressed as cost per congenital syphilis (CS) case averted.

Base case and minimum and maximum values for each two-way sensitivity analysis are as follows, presented as “variable (base case; minimum, maximum)”: 1) DALYs associated with stillbirth (4.95; 0,30) AND AO incidence without treatment (0.52; 0.4,0.7). 2) Treatment performance (0.9; 0.7,0.99) AND AO incidence without treatment (0.52; 0.4,0.7). 3) Adult syphilis averted per pregnant case treated (1; 0,1) AND treatment performance (0.9; 0.7,0.99). 4) Percent active syphilis among reactive cases (0.65; 0.5, 0.8) AND AO incidence without treatment (0.52; 0.4,0.7).
