# Supplementary material for: The Cost and Cost-Effectiveness of Scaling up Screening and Treatment of Syphilis in Pregnancy: A Model
Source: PLoS One. 2014 Jan 29;9(1):e87510. doi: 10.1371/journal.pone.0087510 (PMC3906198; doi:10.1371/journal.pone.0087510)
Supplement: Text S1 — Calculation of DALYs averted by preventing the horizontal spread of adult syphilis and by preventing syphilis-attributable HIV cases. (DOCX) [file pone.0087510.s005.docx]

**Text S1 – Calculation of DALYs averted by preventing the horizontal spread of adult syphilis and by preventing syphilis-attributable HIV cases**

We assume that each case of treated syphilis in pregnancy averts 1 case of adult syphilis. Syphilis is a sexually transmitted infection, and infection with syphilis increases the risk of HIV transmission and acquisition [[1](#_ENREF_1),[2](#_ENREF_2)]. We assume that each case of syphilis averted reduces the risk of new HIV infection, by lowering the prevalence of syphilis as a co-factor for HIV transmission (both to and from the woman treated). We are conservative, assuming just 20% of the benefit of full removal of the risk factor due to clinical trials that suggest that STI treatment is inefficient as a means to reduce HIV risk [[1](#_ENREF_1)]. We consider that treated syphilis in the pregnant woman contributes one-half of an averted syphilis case to the reduction in HIV risk.

We calculated the number of HIV cases averted per case of syphilis in pregnancy treated as follows:

*# SSA x (HIVIncid_STI_ − HIVIncid_NoSTI_) x Effect_Adj_ x Index_adj_*

Where:

***# SSA*:** # secondary syphilis cases averted per case of treated syphilis in pregnancy = 1 (as described above)

***HIVIncid_STI_*:** HIV incidence in the absence of other STIs = 0.0013 per 100 susceptibles per year (author calculation based on an assumed adult HIV prevalence of 0.5%)

***HIVIncid_NoSTI:_*** HIV incidence in the presence of other STIs = 0.0038 (i.e., an assumption of 3 times the incidence in the absence of other STIs)

***Effect_Adj:_*** Effectiveness adjustment = 0.20 (i.e., 20% reduction of the influence of syphilis as a co-factor for HIV transmission)

***Index_adj_*:** Index case adjustment = 1.5 (i.e., one case of treated syphilis in pregnancy contributes one-half of an averted syphilis case to the reduction in HIV risk).

Therefore, we estimated that each case of treated syphilis in pregnancy averts 0.001 cases of HIV.

The disability weights associated with a case of primary, secondary and tertiary syphilis are estimated at 0.015, 0.048, and 0.283, respectively [[3](#_ENREF_3)], with estimated mean durations of each stage (assuming untreated syphilis) of 0.5, 1, and 3 years. In the absence of screening and treatment, the course of syphilis and the rate of mortality are uncertain. We assume untreated tertiary syphilis results in premature mortality (10 DALYs) that occurs 15 years after the onset of primary syphilis. Using a discount rate of 3% per year, we calculated the discounted, cumulative DALY burden associated with untreated adult syphilis to be 6.09 DALYs per case, and estimated the burden from a case of HIV at 7.2 DALYs [[4](#_ENREF_4)]. We then calculated the weighted mean DALYs from adult syphilis and HIV as 0.759 DALYs per STI case. Finally, we estimated the overall DALYs averted due to reduced STIs (syphilis and HIV) in adults as the product of the proportion of MTCT of syphilis averted, the number of STIs averted per MTCT of syphilis averted, and the number of DALYs per STI case.

***Sensitivity analysis of syphilis cases averted per case treated***

In the base case, we assume that each case of syphilis treated in pregnancy averts one case of adult syphilis. If instead we adopt more conservative assumptions, i.e., that each treated case averts 0.5 cases or no cases of adult syphilis, our findings are somewhat less favorable but qualitatively similar: expanded syphilis screening and treatment remains cost-saving in each of the four high prevalence scenarios, and “highly cost-effective” (at less than the per-capita GDP of any country [[5](#_ENREF_5)]) in the remaining four scenarios. Results are shown here:


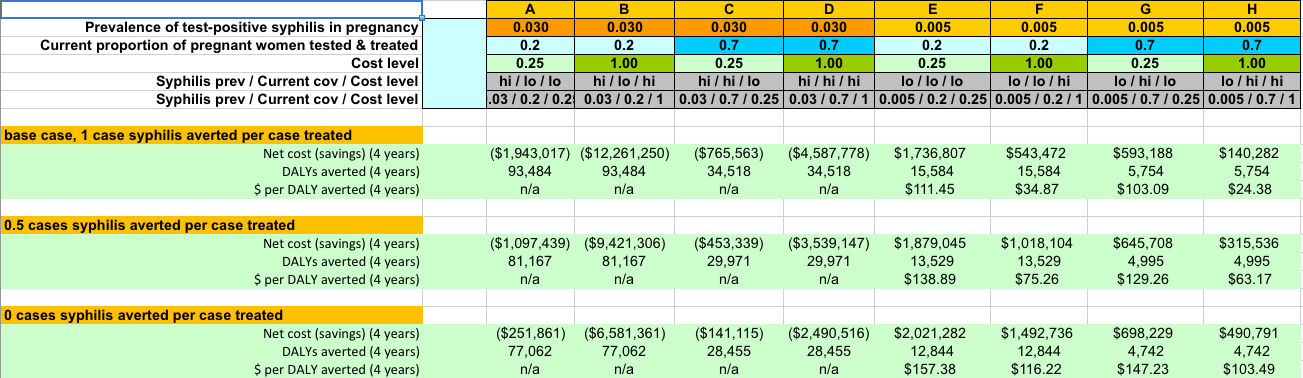
 In the base case, we also assume that untreated tertiary syphilis results in premature mortality 15 years after the onset of primary syphilis. However, since the exact course of syphilis and rate of mortality in the absence of treatment are uncertain, a modified assumption of zero cases of syphilis averted per case of syphilis treated also allows us to examine the effect of treatment assuming no DALY benefits from reduced syphilis transmission. When we reduce or zero out additional cases of syphilis averted, we also indirectly and proportionally reduce or zero out HIV infections averted due to reduced syphilis prevalence.

**References**

1. Fleming DT, Wasserheit JN (1999) From epidemiological synergy to public health policy and practice: the contribution of other sexually transmitted diseases to sexual transmission of HIV infection. Sexually transmitted infections 75: 3-17.

2. Mehta SD, Ghanem KG, Rompalo AM, Erbelding EJ (2006) HIV seroconversion among public sexually transmitted disease clinic patients: analysis of risks to facilitate early identification. J Acquir Immune Defic Syndr 42: 116-122.

3. Lopez AD, Mathers CD, Ezzati M, Jamison DT, Murray CJL (2006) Global Burden of Disease and Risk Factors. Disease Control Priorities Project. Washington (DC): World Bank.

4. Marseille E, Kahn JG, Pitter C, Bunnell R, Epalatai W, et al. (2009) The cost effectiveness of home-based provision of antiretroviral therapy in rural Uganda. Applied health economics and health policy 7: 229-243.

5. The World Bank (2010) GDP per capita (current US$). World DataBank.
